# Hydrophilic Natural Polymers for Sustained-controlled Release of Calcium Hydroxide

**DOI:** 10.22037/ijpr.2019.13447.11623

**Published:** 2020

**Authors:** Negin Farhadian, Mostafa Godiny, Ali Mansouri, Sajad Moradi, Ahmad Tajehmiri, Mohsen Shahlaei

**Affiliations:** a *Substance Abuse Prevention Research Center, Kermanshah University of Medical Sciences, Kermanshah, Iran. *; b *Department of Endodontics, School of Dentistry, Kermanshah University of Medical Sciences, Kermanshah, Iran. *; c *Nano Drug Delivery Research Center, Kermanshah University of Medical Sciences, Kermanshah, Iran. *; d *Medical Biology Research Center, Kermanshah University of Medical Sciences, Kermanshah, Iran.*

**Keywords:** Aloe vera, Calcium hydroxide, Gelatin, Gum tragacanth, Sustained release

## Abstract

Calcium Hydroxide (CH) is commonly employed as intracanal medicament in endodontics. In order to maximize its therapeutic effects, it is essential to develop new approaches for preparing the controlled drug release systems which, in turn, facilities the dissociation of CH into calcium and hydroxyl ions. This work studies the sustained-controlled release of calcium ions and the effect of pH changes on the different formulation of CH with hydrophilic natural polymers over a period of 30 days. Various formulations were prepared by combining CH with gelatin, *aloe vera* and *gum tragacanth*. Root canals of 60 human teeth were instrumented and filled with a different formulation of CH and suspended in plastic tubes containing distilled water. Three formulas of polymer/CH were evaluated, and pure CH powder was used as a control. At specific time intervals, the calcium ions release and the pH changes of the medium in different formulations were analyzed. The main interactions between the studied polymers and CH were investigated using FTIR spectra. The antibacterial activity of formulations against *Enterococcus faecalis* was also studied. Faster release of CH was observed for *aloe vera*/CH. *Gum tragacanth*/CH showed a slow-release during the first 15 days. In contrast, only Gelatin/CH formulation showed a prolonged release with statistically significant differences (*P < *0.05). The pure CH showed significantly higher pH values than the other formulations. The Gelatin/CH formulation was a better sustained-release system than the pure CH, and it can be used as a promising vehicle for CH in the root canal treatment.

## Introduction

Calcium Hydroxide (CH) is often applied in dentistry because of its antibacterial activity, ability to promote mineral hard tissue formation and inhibition of tooth resorption (1, 2). In order to improve the antibacterial properties and the ability to easy handling of CH, its powder is regularly mixed with various chemicals to obtain different formulations for clinical use (3). Commonly different types of systems including aqueous, viscous and oily are reported for CH delivery. The dissociation of CH medicament into hydroxyl and Calcium ions is affected by type of the vehicles applied. In one hand, fast release of ions is the disadvantage of viscous and aqueous vehicles and on the other hand, the application of oily vehicles is limited because of immunogenicity, cytotoxicity, low CH loading, and difficulty in removal (4). An ideal vehicle should have the following properties: allow for gradual-slow calcium and hydroxyl ions release, allow for slow diffusion in the tissues along with low solubility in tissue fluids, and have no harmful effect on the induction of hard tissue deposition (5). According to Han *et al.* (3), sustained release of CH is mainly needed when it is used as a pulp capping agent and intracanal dressing in cases of severe periapical lesions, apexification procedures, and root resorption. Therefore, a vehicle that provides sustained prolonged release of CH with suitable properties (as mentioned above) is deeply needed. 

There are many studies which focused on the preparation of various calcium hydroxide formulations using synthetic and natural polymers to obtain CH-loaded controlled release systems as well as increase the biological performance of CH medicament (3, 4 and 6-8). In this regard, polylactic acid (PLA) (5), ethylcellulose (EC) (2), propylene glycol, poly-ethylene glycol 6000, chitosan, and guar gum (6) were applied as matrices for CH delivery. Moreover, nowadays there is a growing interest in the use of different hydrophilic natural polymers for designing drug delivery systems which are appropriate for retarding drug release (9). Similarly, in recent years, application of natural health remedies as vehicles for drugs and medicaments in dentistry has attracted many attentions (10).

The *aloe vera* gel consists of a liquid phase (99.5% water), and a solid phase, including soluble sugars, enzymes, lipids, proteins, polysaccharides and lignin, vitamins, minerals (*e.g.* calcium, potassium, sodium, magnesium) and phenolic compounds (11). Healing properties of the *aloe vera* gel include anti-inflammatory, antiseptic and antimicrobial activity, which can be particularly important for wound healing applications (12). *Aloe vera* can be used in dentistry in the treatment of different disease such as lichen planus, recurrent aphthous stomatitis, oral submucous fibrosis, alveolar osteitis, periodontitis, *etc.* (13). It has been used as a sedative dressing and file lubrication during biomechanical preparation in root canal treatment (14). Furthermore, Pereira *et al.* reported that the thin hydrogel films composed by alginate and *aloe vera* gel have the light transmission and transparency properties which is suitable for endodontic drug delivery applications (15).

Gelatin, a naturally multifunctional biopolymer, is the digested product of collagen and its structural building blocks are then the amino acids. It is biodegradable, biocompatible, non-antigenic, and nonimmunogenic (16). Gelatin has been extensively investigated as a drug delivery system for many classes of drugs due to its properties as a natural biomaterial and history of safe use in a wide range of medical and pharmaceutical applications (17, 18). 

*Gum tragacanth* (GT) is a branched anionic polysaccharide with properties such as moisture absorption, water solubility, hydrocolloid formation, drug holding, and releasing abilities (19). This natural polymer is a mixture of two soluble and insoluble polysaccharides. Tragacanthin, a branched and water-soluble and an insoluble bassorin, which is a complex of methoxylated acids that swell to form a gel or viscous solution (19). GT besides other synthetic polymers has been used for application in skin regeneration, drug delivery applications, and periodontal defect regeneration (19-21).

For the first time in this study, hydrophilic natural polymers including gelatin, *aloe vera*, and *gum tragacanth* are used to prepare CH formulation. The main goal of this study is to assess the sustained release of calcium ions from biodegradable drug delivery systems loaded with CH. For this purpose, three formulations of gelatin/CH, *aloe vera*/CH, and *gum tragacanth*/CH were prepared and pure CH powder was used as control. The calcium ion release and changes in pH were measured up to 30 days and the antibacterial activities of different formulations were also analyzed. 

## Experimental

*Aloe vera* powder at research grade, 98%, was provided from Besure Healthcare private limited B-257, Okhla Industrial Area phase-1 New Dehli. Gelatin, calcium hydroxide and glycerin were purchased from Sigma-Aldrich (USA). *Gum tragacanth* used in the present study was a ribbon type, gathered from the stems of Fluccosus species of Astragalusbushes, grown in the western areas of Iran.

*Preparation of polymer/CH Gel*

A sustained-controlled release gel formulation was prepared using different natural polymers. Hydrophilic polymers including gelatin, *aloe vera*, and *gum tragacanth* were used for the preparation of CH gel in order to prolong the release. Different concentrations of natural polymers were applied to obtain the desired release of calcium ions from polymer/CH formulation. Preliminary studies were carried out to optimize the gel formulation with different naturally hydrophilic polymers. To prepare the formulation of *aloe vera* and *gum tragacanth*, *aloe vera* and *gum tragacanth* powders were mixed with glycerin under stirring. In the case of *gum tragacanth* and *aloe vera* polymers, when water is used as the solvent, it causes the ionization of CH. These free calcium ions in the medium produce an aggregation, leading to the early formation of polymer plaques. Polymer/CH formulations were prepared using different polymer: CH weight ratios: 1:0.5, 1:1, 1:1.5 and 1:2. Three formulations were obtained with a certain amount of gelatin (25%), *aloe vera* (12%), and *gum tragacanth* (8%). Two substances were mixed using magnetic stirrer until the homogenous distribution of CH was achieved in the gel matrix. For the formulation of gelatin, it was mixed with distilled water under stirring and simultaneously heated to form a gel base. 

*Drug content and Encapsulation efficiency measurement*

To determine the encapsulation efficiency and drug content of the polymer/CH formulations, these formulations were dissolved in 1 M NaOH. Then, the calcium concentration was determined using the measurement of the absorbance of the samples by an Agilent UV–Vis system at 570 nm. For this purpose, a calcium colorimetric assay (O-Cresolphthalein Method) kit (Pars-Azmoon, Iran) was added for 5 min to react with calcium ions which caused violet color in the samples, and then the absorbance of the samples was recorded. The drug content (%) and encapsulation efficiency (%) of the polymer/CH formulations were calculated as follows:

$\mathrm{Drug content\%}=(\frac{\mathrm{Weight of CH in formulation}}{\mathrm{Weight of formulation}})\times 100$

$\mathrm{Encapsulation efficiency\%}=(\frac{\mathrm{Actual CH loading}}{\mathrm{Theoretical CH loading}})\times 100$

*Swelling index study*

*The swelling* behavior of the *polymer/CH formulations* was also investigated. To ensure complete swelling, formulations were allowed to swell for 24 h in a phosphate buffer solution at pH of 7.4. Extra buffer present on the surface of the swelled samples was wiped with the soft tissue paper. The swelling index was determined as:

$\mathrm{Swelling Index}=\frac{\mathrm{Final weight of sample}-\mathrm{Initial weight of sample}}{\mathrm{Initial weight of sample}}$

*Evaluation of the polymer/CH gel formulation*

Sixty single-rooted mandibular premolar teeth were selected for this study. The crowns of all the teeth were removed in a cutting machine, and the length of each tooth was standardized to 15 mm. The working length of the root canal was measured with inserting a number 10 K-type file (MANI) until it was just seen at the apex and then subtracting 1 mm from its length. The canals were prepared by rotary instrumentation with a circumferential filing technique up to size 45 apically. The canals were irrigated with 2 mL of 1% sodium hypochlorite solution using steel 27-Gauge beveled needle. The smear layer was removed by irrigating the canal with 2 mL of 17% EDTA solution and then the root canal was flushed with 5 mL of distilled water to remove any precipitate of EDTA.

Roots were randomly divided into 4 groups, and the root canals were filled with the following intracanal medications:

Group 1 

(n = 15) – *aloe vera*/CH gel formulation

Group 2 

(n = 15) – *gum tragacanth*/CH gel formulation

Group 3 

(n = 15) – gelatin/CH gel formulation

Group 4 

(n = 15) – control, pure CH paste.

*Measurement of the pH and calcium ion release*

The CH release from matrices was measured by using a diffusion membrane for all three formulations. For this purpose, determined amounts of different polymer/CH formulations were inserted into the root canals using K-files (MANI) until it passed the apical foramen. Then, the coronal access cavities were sealed by temporary restorative material and made impermeable using petroleum jelly to prevent it from the moisture contamination. Then, the teeth were immersed in individual plastic tubes containing 10 mL of distilled water with the help of molding wax so that only the apical third of the roots were soaked in distilled water. Each tube was sealed with parafilm and stored in an oven at 37 °C until the established analysis time. After that, specimens of 3.0 mL of the distilled water were withdrawn at predetermined time intervals (1, 7, 15, 21 and 30 days) and subjected to pH and calcium ion release measurements and then these samples were replaced with fresh distilled water. The specimens from each experimental time were shaken in a vortex for 7 s and the pH levels were determined using a pH meter. The calcium ion concentration in the medium was determined with an Agilent ultraviolet spectrophotometer at 570 nm. Data were recorded, analyzed, and subjected to statistical analysis using ANOVA and LSD’s test at 5% significance level. The statistical analysis was done by SPSS 16 software.

*Preparation of different formulations for FTIR analysis*

Fourier transform infrared spectroscopy (FTIR) analysis was applied in order to study the structural changes and the qualitative analysis of different formulations. For recording FTIR spectra, using a sampler, a small amount of gel formulations were deposited on AgCl plates and then dried. The FTIR spectra of the samples observed by transmission through dried gel films placed on the on AgCl plates.

*Antibacterial assay*

*Enterococcus faecalis* (ATCC 29212) was obtained from the Iranian Collection of Industrial Microorganisms Center. The antibacterial performance was evaluated by the paper disk diffusion method. Sterile Mueller-Hinton agar plates were inoculated with the* Enterococcus faecalis* *(E. faecalis) *bacterial suspension. Different concentrations of the substances adsorbed on the paper disks were deposited on the surface of the media. Then, all plates were incubated at 37 °C for 24 h. The antibacterial activity of different substances was assessed by measuring the diameter of the inhibition zone around the disk. Each paper disk was impregnated by 0.08, 0.16, 0.32, and 0.64 µg/μL of substances. Ceftazidime (30 μg) was applied as a control.

## Results and Discussion

*Drug release from three different formulations*

Table 1 shows the Encapsulation Efficiency (EE%), Drug Content (DC%), and swelling index for the best polymer/CH formulations. The systems containing gelatin/CH ratio of 1:1, *aloe vera*/CH, and *gum tragacanth*/CH ratio of 1:2 were found to be the most appropriate according to the encapsulation efficiency and drug content. As presented in Table 1, the encapsulation efficiency of the polymer/CH formulations varied from 76 to 92%. In this case the Gelatin formulation shows better results in term of balance between EE% and DC%. 

In the case of release kinetic, the *aloe vera* formulation because of its very hydrophilic and loose network structure, facilitates leach out of CH and has the fastest drug release than the other two polymeric systems. Determination of the swelling index for different formulations was performed to explain the observed results of the CH release with regard to the rates of polymers hydration. 

**Figure 1.**
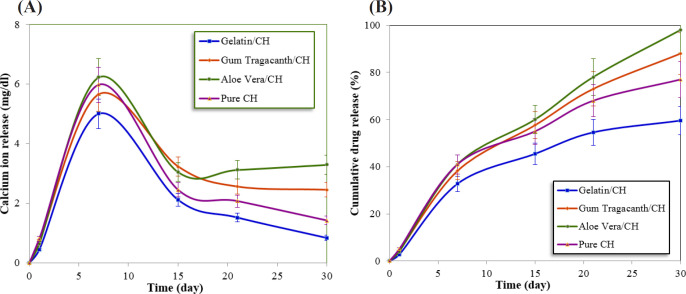
(A) The calcium ion release profile in the 4 groups. (B) The cumulative release of calcium ion for the 4 groups

Tables 2 and 3 represent the means and standard deviations for the calcium ion release (mg/dL) and changes in pH of the formulations in the different studied time periods, respectively. The comparison of mean calcium ion release (mg/dL) with different formulations was assessed using analysis of variance (ANOVA) and LSD test, which is shown in Figure 1A. Figure 1B shows the cumulative release rate of CH in the 4 groups.

The results obtained by ANOVA for both calcium ion release and changes in pH showed significant interactions between formulations and experimental time periods. The hydrophilic nature of polymers in the polymer/CH formulations can significantly facilitate water uptake and swelling of the polymeric matrix. So, faster leakage of the calcium out of the polymeric matrix occurred, resulting in higher release rate along the first few days and reaching a plateau after a certain period of time as evident from Figure 1A.

The Gelatin/CH formulation showed a calcium release profile lower than that of the other three formulations during 30 days; also statistically significant differences between the Gelatin/CH formulation and control were found (*P < *0.05).

As shown in Figure 1B, Gelatin exhibited 60% calcium ion release at the end of 30 days. For *aloe vera*/CH formulation, the same amount of calcium ion was released within 15 days and at the end of the time period, it reaches 98%. Pure CH showed fast diffusion behavior than Gelatin/CH and more than 77% of the CH was released within 30 days. A 88% of CH was released within 30 days for *gum tragacanth*/CH formulation.

Among three naturally polymers, Gelatin exhibits more sustained and prolonged release of CH during the time period. For the preparation of the Gelatin/CH formulation, the applied solvent is water while the used solvent of both other polymer/CH formulations is glycerin. CH dissolves slightly in water but more readily in glycerin. This passive process of diffusion is highly dependent upon the concentration of the substance. The higher concentration leads to the faster the rate of diffusion. If the material dissolved in the carrier, its distribution becomes homogeneous and resulted into an increase in the amount of material per unit volume. In the case of material which precipitated in the carrier, diffusion becomes difficult. Glycerin can dissolved more CH than water but cannot hydrolyze it to its active parts (22). 

With decreasing swelling index, the cumulative CH release decreases. Because the least swelling index amount belongs to gelatin/CH, this formulation showed the more sustained-release system. Possibly the cationic calcium ion could entrap in the negative-charged Gelatin due to their attractive electrostatic interaction. From Figure 1 and Table 2, it can be concluded that faster CH release was observed for the case of a formulation having *aloe vera* gel powder when compared with the other three groups. Hence, when *aloe vera* is used alone as retarding material, it was unable to sustain the CH release. 

*Gum tragacanth* could provide a sustained release of the CH up to 15 days approximately. This may due to better hygroscopic compatibility of the CH and polymeric matrix and possibly due to their attractive electrostatic force. Due to the *gum tragacanth* biodegradability, structural and compositional advantages, natural availability, higher resistance to microbial attacks, and low cost, it can be employed as a proper endodontic drug delivery system.

*pH changes during evaluation of three different formulations*

**Figure 2:**
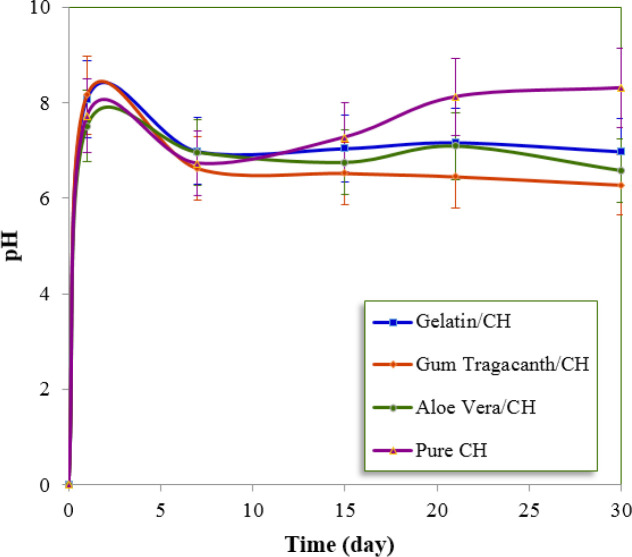
The pH profiles of the 4 groups

The statistically significant difference in pH was found among the different time intervals and also among the different formulations tested. In terms of pH, LSD test revealed that the pure CH system was significantly different from the other formulations in the last three time periods. For 15, 21 and 30 days of the experimental time periods, the highest pH values corresponded to the pure CH, followed by the formulations prepared with gelatin, *aloe vera* and *gum tragacanth*. Figure 2 shows the average pH values of each formulation which were recorded at various time periods. The pH of the three groups (*aloe vera*/CH, *gum tragacanth*/CH, and pure CH) on the first day was found to be approximately 7.0.

Pacios *et al.* have reported that the type of substance added to the CH paste might affect the pH values (7). These reported results are in good agreement with our study. The pure CH showed significantly higher pH values than the other formulations. It should be mentioned that most studies reported pH values between 11 and 12, higher than the findings observed in the present study (23, 24). The reason for this may be that in previous studies, the pH was measured either directly in the CH powder-vehicle mixture or in distilled water after direct putting up the formulation in it. In the present work, the formulations were applied to fill root canals which their coronal access was sealed before immersion in distilled water. During the time period, no increase in the pH of the surrounding media and a non-significant reduction was observed. The buffering capacity of polymers is main key factors affecting the diffusion of hydroxyl ion through root dentin and could explain these findings (7). Most of these formulations show slightly acidic or neutral pH with a mild buffering property that can redox the OH ions to H_2_O_2_. However, the pure CH, obtained by mixing CH powder with the distilled water, has a high pH and promotes a rapid ionic release (25). The 30-day release data were fitted to Zero order, First-order and Higuchi equations and the correlation coefficient values (r) are presented in Table 4. The release kinetics did not fit to the Zero order and first order equations, whereas better results are obtained for Higuchi-square root equation, suggesting that diffusion-controlled transport of CH through the polymer/CH formulations.

*FTIR analysis of three different formulations*

**Figure 3:**
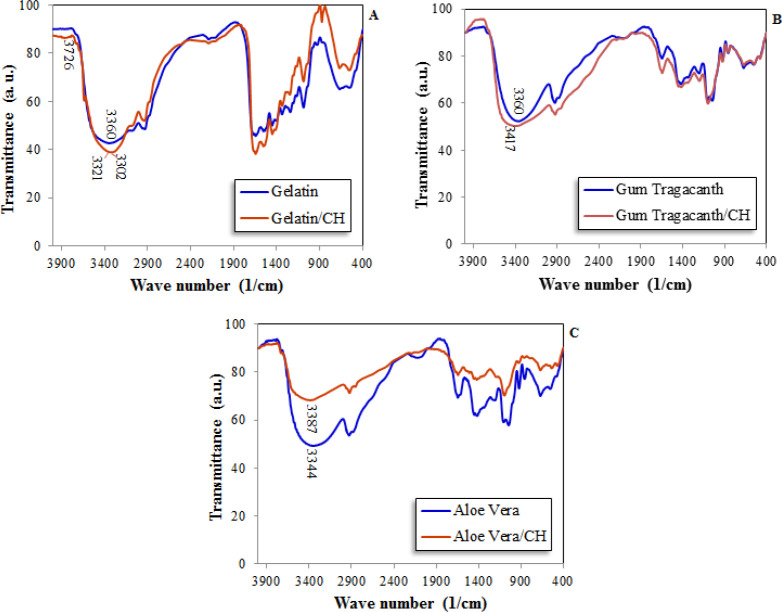
FTIR spectra of (A) Gelatin and gelatin/CH, (B) *Gum tragacanth* and *gum tragacanth*/CH, (C) *Aloe vera* and *aloe vera*/CH

Figure 3 shows FTIR spectra of three different formulations containing polymers and CH and the spectra of polymers without CH are also presented. These spectra were taken in the wavelength ranging between 500 and 4,000 cm-1. 

As shown in the figure, there are no significant differences between the spectra of free and or formulated polymers, and it can be concluded that the formation of new bonds between CH and polymers were not occurred. This finding is important from the two points of view; the release kinetic of CH could not be affected by polymer bonding and is only diffusion controlled, the new polymeric-CH compounds are not formed to cause probable cell cytotoxicity. 

*Antibacterial evaluation*

The use of biocompatible intracanal medicaments with antimicrobial properties between appointments may lessen or eradicate bacteria in the root canal and hence increase the success of root canal treatment (26). All substances (including gelatin, *aloe vera*, *gum tragacanth*, three polymer/CH formulations, glycerin, and CH) were screened for their antibacterial efficacy by agar disc diffusion method. The antibacterial activities of different substances were evaluated by the diameter of the inhibition zone around the disk; these measured diameters are reported in Table 5.

The type of vehicle used may have an important influence on the antimicrobial activity of CH (27). The diameter of the inhibition zones around the disk created by each substance is presented in Table 5. The following scale of measurement: zone of inhibition of >15 mm as strongly inhibitory, 10-15 mm as moderately inhibitory, and <10 mm as not inhibitory was considered for the interpretation of the antibacterial tests. However, CH has excellent antimicrobial properties, for its tested concentrations in the present study; CH was not able to eliminate *E. faecalis* sufficiently. This is consistent with the results of the other reports (28, 29). Results in Table 5 showed that the antimicrobial activity of Gelatin against *E. faecalis* was increased when manipulated with CH and indicating a moderately antimicrobial activity against this microorganism. However, for other substances, a prominent antimicrobial efficacy can’t be seen at any of the studied concentrations. For example, the inhibition zones of *gum tragacanth* and *gum tragacanth*/CH were similar against *E. faecalis*. It is clear that many of the tested materials were found not active against the bacteria as *aloe vera* or *aloe vera*/CH. In a study by Wynn, *aloe vera* gel showed inhibitory effects on *E. faecalis* because of its anthraquinine components (30) and its bactericidal activity is found to be less than that of CH (31).

**Table 1:** Results of encapsulation efficiency, drug content and swelling index measurement

**Type of formulation**	**Encapsulation efficiency (%)**	**Drug content (%)**	**Swelling index**
Gelatin/CH	92 ± 1.27	48 ± 2.53	1.24 ± 0.21
*Gum tragacanth*/CH	88 ± 1.83	63 ± 3.12	1.41 ± 0.19
*Aloe vera*/CH	76 ± 3.22	60 ± 1.56	3.59 ± 0.35

**Table 2:** Means and standard deviations for the calcium ion release (mg/dL) observed at different times

**Period (day)**	**Gelatin/CH**	***Gum tragacanth*** **/CH**	***Aloe vera*** **/CH**	**Pure CH**
1	0.445* ± *0.118	0.717* ± *0.118	0.647* ± *0.118	0.813* ± *0.145
7	5.004* ± *0.393	5.651* ± *0.393	6.231* ± *0.393	5.974* ± *0.482
15	2.114* ± *0.230	3.232* ± *0.230	3.046* ± *0.230	2.448* ± *0.282
21	1.521* ± *0.259	2.558* ± *0.259	3.121* ± *0.259	2.072* ± *0.318
30	0.838* ± *0.375	2.451* ± *0.375	3.291* ± *0.375	1.425* ± *0.459

**Table 3:** Means and standard deviations for pH of the groups observed at different times

**Period (day)**	**Gelatin/CH**	***Gum tragacanth*** **/CH**	***Aloe vera*** **/CH**	**Pure CH**
1	8.085* ± *0.247	8.167* ± *0.247	7.509* ± *0.247	7.729* ± *0.302
7	6.985* ± *0.040	6.627* ± *0.040	6.960* ± *0.040	6.731* ± *0.049
15	7.045* ± *0.122	6.524* ± *0.122	6.749* ± *0.122	7.279* ± *0.150
21	7.169* ± *0.072	6.453* ± *0.072	7.090* ± *0.072	8.123* ± *0.088
30	6.983* ± *0.091	6.278* ± *0.091	6.577* ± *0.091	8.310* ± *0.111

**Table 4.** Release Kinetics of polymer/CH formulations

**Type of formulation**	**Zero order**	**First-order**	**Higuchi-square root**
Gelatin/CH	K_0 _= 0.001 r^*^= 0.58	K_1 _= 0.0077 r = 0.76	K_H _= 0.0517 r = 0.79
*Gum tragacanth*/CH	K_0 _= 0.0014 r = 0.8	K_1 _= 0.0006 r = 0.66	K_H _= 0.0542 r = 0.8
*Aloe vera/*CH	K_0 _= 0.001 r = 0.56	K_1 _= 0.0025 r = 0.87	K_H _= 0.0552 r = 0.8
Pure CH	K_0 _= 0.002 r = 0.89	K_1 _= 0.0004 r = 0.4	K_H _= 0.0522 r = 0.79

^*^r correlation coefficient value, K_0_ represents Zero order release constant, K_1_ is First-order release constant, and K_H_ is Higuchi rate constant.

**Table 5:** Inhibition zone diameter (mm) measured for different substances against *E. faecalis*

**Type of material**	**C** ^+^ ** (µg/µL)**	**ZOI** ^*^ ** (mm)**	**Type of material**	**C (µg/µL)**	**ZOI (mm)**
Gelatin	0.08	7.5	*Gum tragacanth*	0.08	7.5
	0.16	9		0.16	8.4
	0.32	11.5		0.32	9.2
	0.64	12.5		0.64	10
Gelatin/CH	0.08	9	*Gum tragacanth*/CH	0.08	7
	0.16	10		0.16	7.5
	0.32	12		0.32	8.5
	0.64	14		0.64	9.5
*Aloe vera*	0.08	8	CH	0.08	7
	0.16	8.6		0.16	7.25
	0.32	9		0.32	8
	0.64	9.4		0.64	9
*Aloe vera*/CH	0.08	8	Glycerin	0.08	8
	0.16	8.8		0.16	9
	0.32	9		0.32	9.3
	0.64	9.3		0.64	9.8
Ceftazidime	30 µg	22			

^+^Concentration, ^*^zone of inhibition.

## Conclusion

This study presents the new controlled drug release vehicles for CH that is employed as an intracanal medicament in endodontics. The systems were prepared by combining CH with different biomaterials of gelatin, *aloe vera* and *gum tragacanth*. The presence of glycerin in *gum tragacanth*/CH and *aloe vera*/CH formulations caused a faster release of calcium than gelatin/CH which contains water in its structure. Three of the polymer/CH formulations behaved similarly in terms of pH and for 15, 21 and 30 days, the pure CH showed significantly higher pH values than the other formulations. Results show that a hydrophilic polymer drug delivery system loaded with CH (gelatin/CH) might be useful for root canal treatment because this system would release calcium during a long time with a single dose application. Higuchi equation is the best kinetics model to describe the release profile of the CH and hence, the CH release from polymer/CH formulations follows the diffusion mechanism. Results of the FTIR analysis indicate the presence of a hydrogen bond between *gum tragacanth*, gelatin, and CH. Also, according to the antibacterial test results, the gelatin/CH formulation exhibits a moderate antimicrobial activity against *E. faecalis*. In conclusion, considering the sustained release of CH and based on statistical results, the intracanal use of a gelatin/CH can be suggested instead of the traditional distilled water/CH combination. 
